# Supplementary material for: All-Trans Retinoic Acid Attenuates Fibrotic Processes by Downregulating TGF-β1/Smad3 in Early Diabetic Nephropathy
Source: Biomolecules. 2019 Sep 25;9(10):525. doi: 10.3390/biom9100525 (PMC6843855; doi:10.3390/biom9100525)
Supplement: Supplementary file 1 [file biomolecules-09-00525-s001.pdf]

All-Trans Retinoic Acid Attenuates Fibrotic Processes by Downregulating TGF-β1/Smad3 in Early Diabetic Nephropathy

Edith Sierra-Mondragon [1], Rafael Rodríguez-Muñoz [1], Carmen Namorado-Tonix [1], Eduardo Molina-Jijon [2], Daniel Romero-Trejo [3], Jose Pedraza-Chaverri [4], and Jose L-Reyes [1,*]

Supplementary Materials:

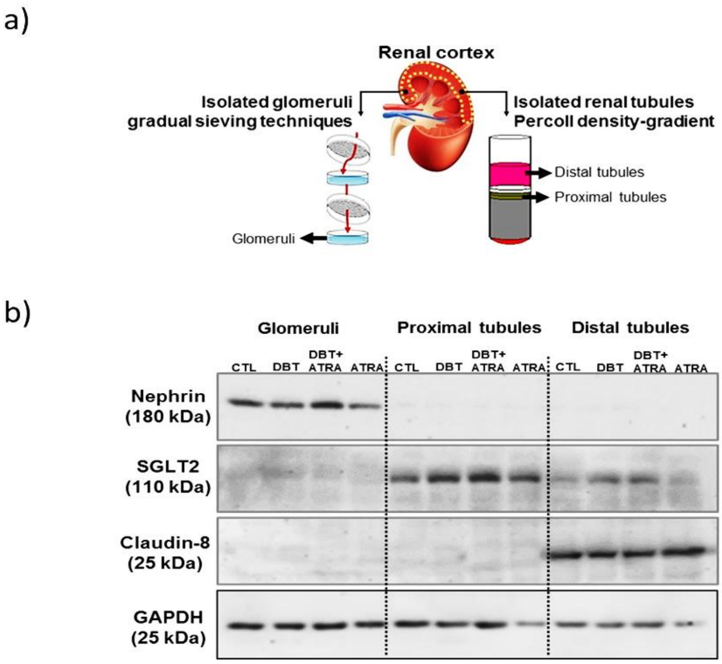

Figure S1. Enrichment of nephron sections. (**a**) Experimental procedure to isolate nephron segments from renal cortex as described in Methods section. (**b**) Specific markers of glomeruli (nephrin), proximal tubules (sodium glucose transporter 2, SGLT2), and distal tubules (claudin-8). Glyceraldehyde 3 phosphate dehydrogenase (GAPDH) was used as loading control. CTL = Control group, DTB = Diabetic group, DBT+ATRA = Diabetic rats treated with all-trans retinoic acid, ATRA = Control animals treated with all-trans retinoic acid.
